# The Effect of Viscosity-Modifying Admixture on the Extrudability of Limestone and Calcined Clay-Based Cementitious Material for Extrusion-Based 3D Concrete Printing

**DOI:** 10.3390/ma12091374

**Published:** 2019-04-28

**Authors:** Yu Chen, Stefan Chaves Figueiredo, Çağlar Yalçinkaya, Oğuzhan Çopuroğlu, Fred Veer, Erik Schlangen

**Affiliations:** 1 Faculty of Civil Engineering and Geosciences, Delft University of Technology, 2628 CN Delft, The Netherlands; S.ChavesFigueiredo@tudelft.nl (S.C.F.); C.Yalcinkaya@tudelft.nl (Ç.Y.); O.Copuroglu@tudelft.nl (O.Ç.); Erik.Schlangen@tudelft.nl (E.S.); 2 Department of Civil Engineering, Dokuz Eylül University, Izmir 35390, Turkey; 3 Faculty of Architecture and the Built Environment, Delft University of Technology, 2628 BL Delft, The Netherlands; F.A.Veer@tudelft.nl

**Keywords:** extrudability, ram extruder, limestone and calcined clay, extrusion-based 3D concrete printing, viscosity-modifying admixture

## Abstract

To investigate the effects of viscosity-modifying admixture (VMA) on the extrudability of limestone and calcined clay-based cementitious materials, three mix designs with different dosages of VMA were proposed in this study. The ram extrusion was utilized as an extrusion model for exploring the fresh properties of printable materials. Two methods were used, based on the ram extruder setup—(a) extruding materials with the same extrusion speed at different rest times to determine how the pressure changes with time; (b) extruding materials with different extrusion speeds at the same rest time to investigate the material flow parameters using the Basterfield et al. model. The main findings of this study could be summarized as—(1) the extrusion pressure of all mix designs exhibited an increasing trend with time. At the same tested age, the extrusion pressure under 0.25 mm/s of piston speed was increased and the shape retention of the extruded filaments was enhanced by increasing the dosage of VMA; (2) the correlation between the experimental results and the Basterfield et al. model was excellent (R-squared value: 0.99). The mixture with a higher content of VMA showed an increased elongational yield stress, flow consistency, and shear yield stress.

## 1. Introduction

**Figure 1:**
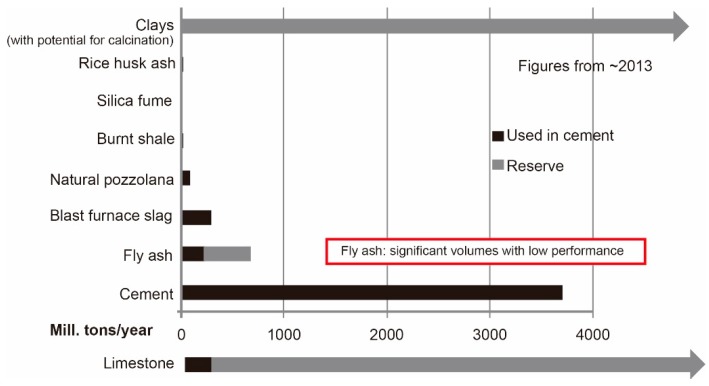
Quantities of common supplementary cementing materials (SCMs). Reproduced from: Scrivener et al. [16].

For the last decade, additive manufacturing (AM) or 3D printing has been significantly developed, worldwide [1]. AM techniques could be applied in the fields of aerospace [2,3], medical treatment, and construction [4]. Extrusion-based 3D concrete printing (3DCP) as a recent implementation showed the potential of using AM techniques as large-scale fabrication methods [5]. Recently, extrusion-based 3DCP has been getting an exponentially increasing attention from both academia and industry. The number of research efforts and innovations on the extrusion-based 3DCP have been growing rapidly [1,6,7,8]. Many advantages of this technique have been demonstrated, such as the elimination of formwork, reducing wastes, increased design freedom, and significant saving in costs and labor [5,9,10,11,12]. However, several issues remain that impede the use of extrusion-based 3DCP in the construction industry. One of the problems is that most of the current printable concrete, contains a relatively higher content of ordinary Portland cement (OPC), in comparison to the conventional concrete [13,14,15]. Sustainability aspects have not yet been prioritized in the development of current printable mix designs. The solution proposed by Chen et al. [14] was to replace a part of OPC in 3D printable mortars, with a combination of limestone and calcined clay, which is generally regarded to be the preferred way for low CO_2_ emissions. The limestone calcined clay cement (LC^3^) which contained 50% of clinker, has been successfully produced by industry trials in Cuba and India [16]. The advantages and sustainability of limestone calcined clay cement were summarized by Scrivener et al. [16], Avet et al. [17], and Antoni et al. [18], including:

- Clays are abundant materials throughout the world. The available amount of clay is much higher than other common supplementary cementing materials (SCMs), such as fly ash, silica fume, and slag (see Figure 1).
- The heating temperature for making calcined clay (700–850 °C) is much lower than for the clinker. A relatively lower amount of CO_2_ is emitted during the producing process of calcined clay, compared with the clinker [19].
- Good, early-age mechanical performance of hardened concrete could be achieved, even by using low-grade calcined clay (contained at least 40% of metakaolin). Up to 50% of the clinker in the binder could be replaced by limestone and low-grade calcined clay, with a comparable compressive strength after 7 days [17].
- The products from the pozzolanic reactions could contribute to a refinement of the porosity [17,18].
- Comparable durability (good resistance to chloride penetration and sulfates attack) [16].

The available mix designs of the limestone calcined clay mortar from the literature could not be directly used in this study. Since there is a unique casting process for the extrusion-based 3DCP, the printable mixture required proper rheological properties for satisfying the demands of extrudability and buildability. In the absence of formwork, the deposited layer should have a sufficient yield stress, to ensure its stable shape and to sustain the increased load from the subsequent layers [20,21]. Many methods have been tailored in the material aspect, to enhance the shape retention and increase the yield stress of printable materials at rest. Adding admixtures might be the most efficient way to manipulate the yield stress evolution. Two groups of additives (thickening and acceleration) were illustrated by Reiter et al. [9]. The viscosity-modifying admixture (VMA) belongs to the thickening additive, which could absorb water and increase the viscosity of solid suspensions. In this study, a Hydroxy Propyl Methyl Cellulose (HPMC)-based VMA was used to promote the flocculation behavior of fresh limestone and the calcined clay-based cementitious materials. Generally, the HPMC-based VMA has been widely utilized to improve the bleeding and segregation of self-compacting concrete [22]. Many side-effects of VMA on cementitious materials, such as prolonged initial setting time, retarded hydration and increased the porosity content, have been demonstrated by Ma et al. [22], Figueiredo et al. [23], and Ou et al. [24]. For rheological properties, the addition of VMA could significantly increase the viscosity of fresh sulphoaluminate cement and mortar [25]. As reported by Figueiredo et al. [6], mixtures with higher dosages of VMA could contribute to a better shape stability of the extruded mortar filaments, but showed a higher extrusion pressure, bulk, and shear yield stress, which might have affected the extrudability. Thus, it was possible to find the optimized VMA content which could not only enhance the shape retention of the extruded mortar filaments but also had less impact on extrudability. However, the effects of VMA dosage on the extrudability of limestone and calcined clay-based cementitious materials, have not yet been investigated.

Extrusion as an efficient, economical, and environment-friendly manufacturing method has been applied in the cement and concrete industries, for producing high performance and fiber-reinforced concrete products [26]. Currently, the ram extrusion is employed as an extrusion model for exploring the extruding properties of printable materials. Perrot et al. [27] pointed out that ram extrusion is a lab-scale method to study rheological behaviors and evaluate the extrudability of different materials. In theory, the rheological behavior of cementitious materials, with the addition of VMA, should be described as elasto-viscoplastic. However, to compare with elastic strain, plastic strain is much higher for most of the materials in the extrusion process. Thus, these materials should also exhibit rigid-viscoplastic behavior [26]. On the other hand, it is feasible to use the ram extruder to assess the fresh properties of very stiff cementitious materials, which could not be evaluated by rotational rheometers [27]. Recently, many researchers including Figueiredo et al. [6], Ogura et al. [8], and Nerella et al. [28] attempted to quantify the extrudability of cementitious materials for extrusion-based 3DCP, by using a ram extruder. Ogura et al. [8] and Nerella et al. [28] evaluated the extrudability of mixtures by measuring the extrusion force. Figueiredo et al. [6] determined the rheological parameters of different solid suspensions, by a ram extruder and the Benbow–Bridgwater model [29] which is the most widely used method for analyzing orifice extrusion data [30]. When the ram extruder has a sharp-edged orifice, the Benbow–Bridgwater equation can be demonstrated as:

(1) $$P=2\left(\sigma_{0}+\alpha V^{n}\right)\ln\left(\frac{D_{0}}{D}\right)$$

where *σ_0_* is uniaxial yield flow stress/elongational yield stress, *α* and *n* are the fitting parameters. Basterfield et al. [30], Perrot et al. [31], and Zhou et al. [26] illustrated that the parameters *α* and *n* in the Benbow–Bridgwater model (Equation (1)) do not belong to the intrinsic material factors. A more advanced and fundamental model was proposed by Basterfield et al. [30]. This model was adapted from the Gibson equation (neglecting the end effect) to characterize the rigid-viscoplastic materials. A spherically convergent flow was assumed and the shear stress in the barrel was ignored. The specific derivation process of the Basterfield et al. model (Equation (2)) was given by Basterfield et al. [30] and Zhou et al. [26].

(2) $$P=2\sigma_{0}\ln\frac{D_{0}}{D}+\frac{2}{3n}k{\left(\sin\theta_{max}\left(1+\cos\theta_{max}\right)\right)}^{n}\left(1-{\left(\frac{D}{D_{0}}\right)}^{3n}\right){\left(\frac{2V}{D}\right)}^{n}$$

In Equation (2), *σ_0_*, *k*, and *n* represent uniaxial yield flow stress/elongational yield stress, flow consistency, and flow index, respectively. Compared with the Benbow–Bridgwater model (Equation (1)), the primary advantage of the Basterfield et al. model (Equation (2)) is that all parameters are physically meaningful, especially the flow consistency *k* [30]. Zhou et al. [26] used this model to investigate the rheological properties of short fiber-reinforced semi-solid fresh cement mortars. Perrot et al. [31] illustrated that the ram extrusion is also influenced by the material tribology. The authors developed a novel ram extruder and extended the Basterfield et al. model by adding the contribution of shear stress, on the tapered surface [27].

On the other hand, as explained by Basterfield et al. [30] and Perrot et al. [27], the cementitious materials could be considered to follow the Von–Mises criterion. Therefore, the analytical relationship between the elongational yield stress *σ_0_* and the shear yield stress *τ*_0_ are related, as described in Equation (3).

(3) $$\tau_{0}=\frac{\sigma_{0}}{\sqrt{3}}$$

This paper aimed to investigate the effects of VMA on the extrudability of limestone and calcined clay-based cementitious materials. In this paper, three mix designs with different dosages of VMA were proposed to perform the extrudability tests. Two methods were conducted, based on the ram extruder setup—(a) a method described in Chen et al. [32] was initially used to determine how the extrusion pressure changes with time. The extruded filaments at different ages were also evaluated by the visual inspection. (b) Another method used the extruding material with different velocities at the same rest time, to investigate the material flow parameters, including elongational yield stress, flow consistency, and flow index, by using the Basterfield et al. model [26,30]. Afterward, the shear yield stress was calculated using the Von–Mises criterion. Finally, the test results and further research work for exploring the optimal VMA dosage of limestone and calcined clay cementitious materials for 3DCP, have been discussed and summarized.

## 2. Materials and Methods

### 2.1. Material and Mix Design

**Figure 2:**
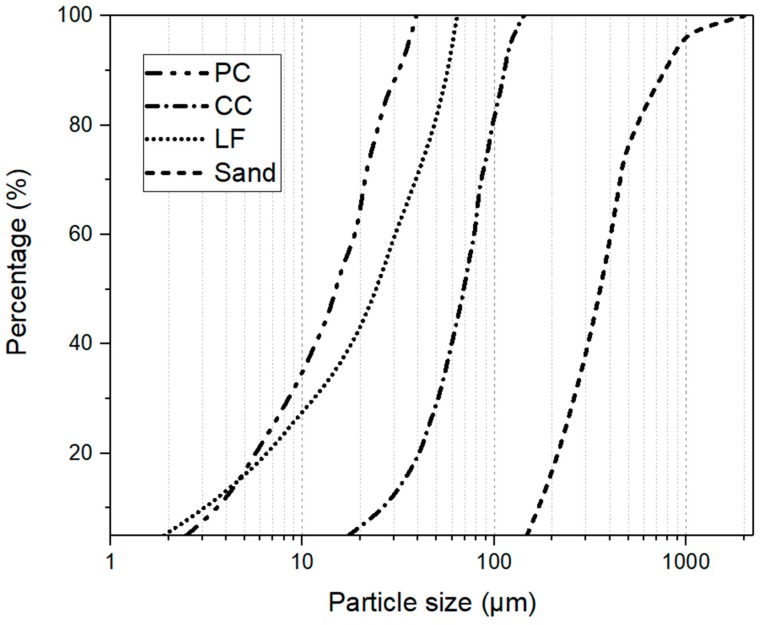
Particle size distribution analysis of dry components.

CEM I 52.5R Portland cement (PC) with 17.9 µm of average particle size (D50) was chosen in this study. A calcined clay (CC) containing about 40 wt.% of metakaolin was sourced from Argeco, France (D50 = 69.35 µm). The limestone filler (LF) with an average particle size of 24.19 µm was also selected. As shown in Table 1, the chemical composition of the powder raw materials was analyzed by X-ray fluorescence spectrometry (XRF, Epsilon 3 XLE EDXRF Spectrometer, Malvern Panalytical, Westborough, MA, United States). Additionally, sand with a maximum grain size of 2 mm was used as aggregates. Figure 2 illustrates the particle size distribution of all dry components in this study.

According to the studies by Avet et al. [17] and Antoni et al. [18], limestone-to-calcined clay ratio of 1:2 showed the best compressive strength during the first 28 days of curing. In this case, the binder was designed as 40% of PC, 40% of CC, and 20% of LF. Based on previous extrusion tests, the suitable binder to sand mass ratio was 1:1.5 and the water to binder mass ratio was selected as 0.3. To adjust the fresh properties of the mix designs, a polycarboxylate ether-based superplasticizer (SP), and a Hydroxy Propyl Methyl Cellulose (HPMC)-based VMA were used. About 2% (of the binder mass) of SP was used to ensure the workability of the fresh matrix. Antoni et al. [18] have proved that up to 2% of SP is feasible to be used in limestone and calcined clay-based cementitious materials with small impacts on the hydration process. Adding a small dosage of VMA could improve the cohesion, water-retaining property, and buildability of the printable cementitious materials [6,33]. VMA was also used in cementitious materials for preventing water drainage during the extrusion process [27]. As illustrated in Table 2, the VMA dosage was the main parameter of the different mix designs. The 28 days compressive strength (casted samples—40 mm cube) of MIX-2 was 33.9 ± 1.9 MPa.

For preparing the fresh materials in the extrusion tests, the specific mixing procedures were given as follows:

- Homogenize the dry components by a planetary mixer (HOBART N50, HOBART, Offenburg, Baden-Württemberg, Germany), for 4 min at a low speed (60 rpm).
- Add the water-based solution (water and superplasticizer) while mixing at a low speed.
- After 2.5 min, pause, scrape the walls and blade (a dough-like mixture is generated).
- Mix the mixture again at a higher speed (124 rpm) for 1.5 min.
- Stop, start to fill the barrel.

The time zero (t = 0) in this paper was defined as the time of adding the water-based solution to the dry components.

### 2.2. Ram Extruder and Test Procedure

#### 2.2.1. Ram Extruder

**Figure 3:**
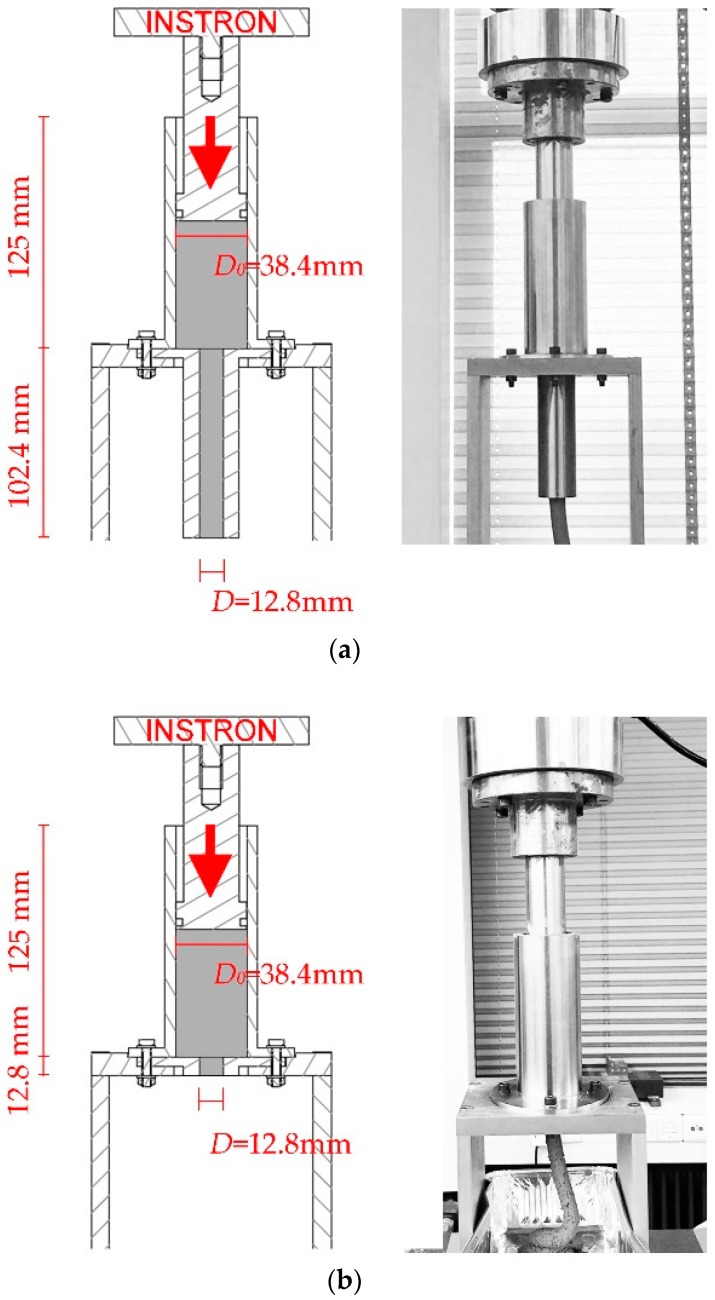
(**a**) Schematic section drawing of the ram extruder with a long die and photograph of the ram extruder with a long die; and (**b**) schematic section drawing of the ram extruder with a short die and photograph of the ram extruder with a short die.

The design of the ram extruder was based on reports by Zhou et al. [26] and Benbow and Bridgwater [34]. As shown in Figure 3, the ram extruder consisted of four stainless-steel components—a piston (Fluon/polytetrafluoroethylene ring on the bottom of the piston), a barrel (the internal diameter: *D_0_* = 38.4 mm), a stand, and a die (the internal diameter: *D* = 12.8 mm). The entire setup was built on a servo-hydraulic press machine (Instron 8872, INSTRON, Norwood, MA, United States), which applied the extrusion force. The piston of the ram extruder was connected with the 10 kN load cell of Instron 8872, in order to measure the total extrusion force. To perform the test of extrusion pressure at different rest times, a long die of length 102.4 mm was used (see Figure 3a). Using a long die could contribute to forming stable shapes of the extruded mortar filaments, which might be regarded as evidence for evaluating the extrusion quality. According to Perrot et al. [31], the friction appeared in the internal surface of the barrel and the die, during the extrusion process. Shortening the length of the die could reduce the frictional effects. A short die (12.8 mm) (see Figure 3b) which could be regarded as a near sharp-edged orifice was used for determining the test of the extrusion pressures, with different extrusion rates. In this study, the authors assumed a pure slip condition at the inner wall of the ram extruder which was an ideal setting. The shear stress between the tested material and the ram extruder was not taken into consideration.

#### 2.2.2. Extrusion Pressures with Different Ages

**Figure 4:**
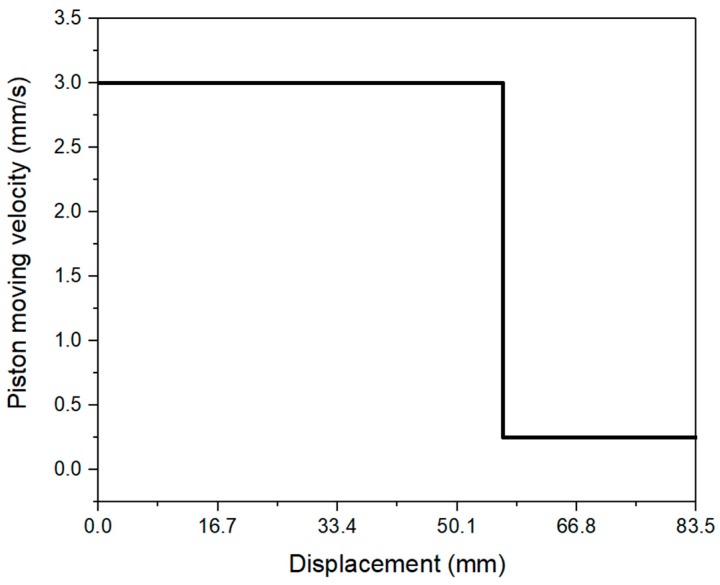
Predefined piston speeds and displacements for the test of extrusion pressure with different ages.

**Figure 5:**
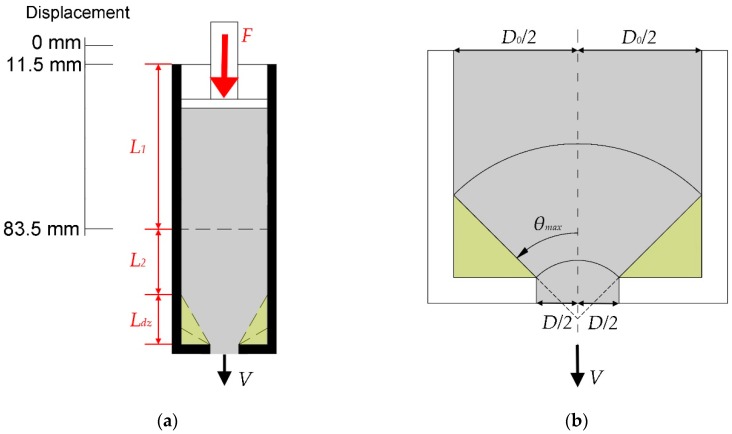
(**a**) Schematic diagram of the barrel section, *L_1_* is the maximum piston moving depth, *L_dz_* is the length of dead zone, *L_2_* is the distance between the piston moving area and the dead zone; (**b**) illustration of the die entrance flow region in a spherical coordinate system, based on Basterfield et al. [30] and Zhou et al. [26]. Yellow areas represent the static zone in the barrel during the extrusion test.

After the mixing process, about 0.16 L of fresh material was filled into the barrel and die were cast by hand. A sealed plastic bag was used to collect the remaining materials. Before performing the extrusion test, the surface of the piston and the Fluon ring were lubricated by a silicone release compound (Dow Corning 7 Release Compound), to minimize the friction between the piston and barrel. The entire test was performed, based on a measurement protocol. The piston displacement was predefined to a fixed distance, at a constant speed. As shown in Figure 4, the piston moved with a speed of 3 mm/s from the displacement of 0 to 56.5 mm (pre-test zone) initially. Then, 0.25 mm/s of piston speed was occupied in the measuring zone (displacement from 56.5 to 83.5 mm), until the end of the first test. The extrusion force (*F*) under 0.25 mm/s piston speed, was collected as the test value. As demonstrated by Perrot et al. [31], when the piston approached the dead zone area, the extrusion force would be increased excessively. The static/dead zone was illustrated in the yellow areas of Figure 5a,b. *θ_max_* represents the maximum convergent flow angle (see Figure 5b) which has been indicated by Basterfield et al. to be in the range of 40–60 degree for flow of most paste-like materials [30]. The length of the dead zone *L_dz_* in the barrel (see Figure 5a) could be calculated by Equation (4):

(4) $$L_{dz}=\frac{\left(D_{0}-D\right)}{2\tan\theta_{max}}$$

where *D_0_* is the inner diameter of the barrel and *D* is the inner diameter of the short die. The maximum value of *L_dz_* is about 21.2 mm (when *θ_max_* equals to 40 degrees). As shown in Figure 5a, the predefined maximum penetration depth of the piston *L_1_* was 72 mm in this study. Thus, there was a distance *L_2_* (at least 31.8 mm) between the piston and the dead zone area, during the test. Influences of the dead zone for the extrusion force could be minimized.

For one mix design, the fresh material was tested under this measurement protocol at different ages (t = 10, 25, 45, 60, 90 and 120 min). According to the Vicat test (setting time test) results, the initial setting times of mixtures MIX-1 (129 min), MIX-2 (147 min), and MIX-3 (161 min) were more than 120 min. Since it took about 2 min to perform one experiment, the tested material could remain homogenous during the extrusion process. After each experiment, the piston and Fluon ring were removed and then cleaned using tap water and soap. The average results were achieved by three repeated experiments of each mix design. A high-resolution camera was used to take photos of the final extruded filaments. The quality of the extruded filaments was assessed by visual inspection. All tests took place under the same ambient conditions (20 ± 2 °C, 55% RH). The upstream extrusion pressure could be determined as:

(5) $$P=\frac{4F}{\pi D_{0}^{2}}$$

where *P* is the extrusion pressure and *F* is the average extrusion force, under one predefined piston moving velocity.

#### 2.2.3. Extrusion Pressures with Different Material Flow Rates

**Figure 6:**
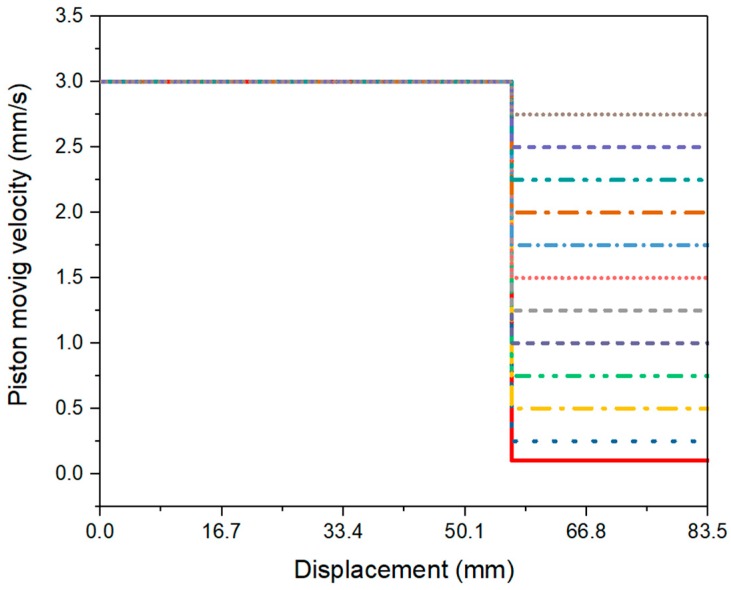
Predefined piston speeds and displacements for the test of extrusion pressure with different material flow velocities.

The general test procedure was very similar to Section 2.2.2; only two differences existed. First, the fresh material from each batch was only tested once, at the age of 10 min. Second, as shown in Figure 6 and Table 3, 12 different piston speeds in the range of 0.10 to 2.75 mm/s were performed in the measuring zone. Thus, it required at least 12 batches and 12 experimental trials for one test. To determine the average result, three repeated tests were conducted for each mix design. The extrusion pressure *P* under different piston speeds could be calculated by Equation (5). Table 3 demonstrates the piston speeds *V_0_* and specific material flow rates *V* at the orifice, which was not measured directly. As explained by Zhou et al. [26], the values of *V* were calculated, based on the assumption that highly viscous cementitious materials are incompressible during the extrusion process. To investigate the analytical relationship between the extrusion pressures *P* and material flow rates *V* at the orifice, the Basterfield et al. [30] model (see Equation (2)) was used. In this case, 45 degrees was selected as the value of *θ_max_* which was recommended by Basterfield et al. [30], Zhou et al. [26], and Perrot et al. [31].

## 3. Results and Discussion

### 3.1. Extrusion Pressures with Different Ages

**Figure 7:**
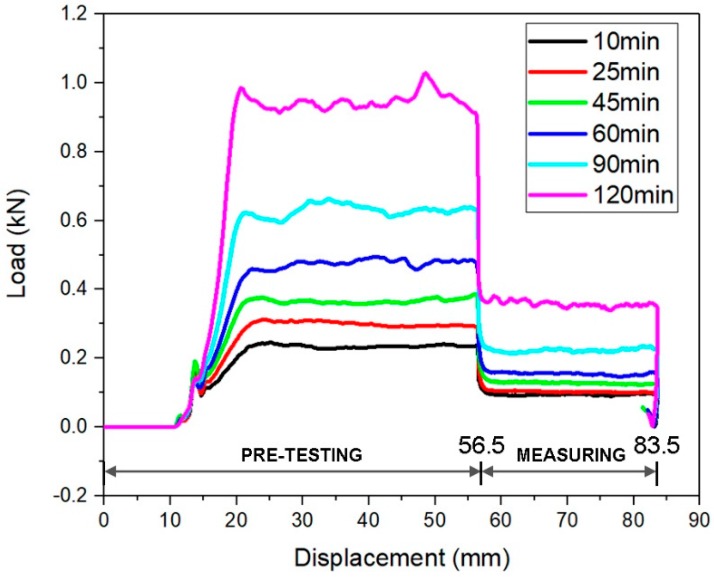
A typical plot of the test—extrusion pressures with different ages (MIX-2).

An example of the extrusion force with respect to piston displacement is illustrated in Figure 7. The displacement from 0 to 56.5 mm belonged to the pre-testing zone, which was used to compact the tested material. Additionally, the thixotropic properties of fresh mixtures were quite significant, due to the presence of VMA. As reported by Marchon et al. [35], the fresh printable cementitious materials might show different rheological properties before and after deposition from the nozzle. Static yield stress is much larger than the dynamic yield stress [36]. Roussel [21] gave a sufficient explanation about this phenomenon from a microstructural point of view. To investigate the extrudability of the mixture, the fresh cementitious material should be kept in a dynamic state, to determine the extrusion force. The extrusion force was collected in the measuring zone. For all curves in Figure 7, the extrusion force remained zero, during the displacement from 0 to 11.5 mm, as the piston had not touched the tested material yet. Once the piston was inserted into the barrel, the extrusion force increased immediately and reached a peak force at the approximate displacement of 21.5 mm. The extrusion force remained at a relatively steady state, until the end of pre-testing zone (displacement of 56.5 mm). During the extrusion process in the pre-testing zone, many unexpected small increases and drops, due to the air voids and hardened particles of the tested material, frequently appeared at the older ages (t = 60, 90, and 120 min). Afterward, the piston speed decreased to 0.25 mm/s, and all curves reached near even state in the measurement zone (displacements between 56.5 and 83.5). The pattern of those curves was not similar to the results obtained by Ogura et al. [8], Nerella et al. [28], and Perrot et al. [31], since a different ram extruder setup and measurement protocol were employed in this study.

**Figure 8:**
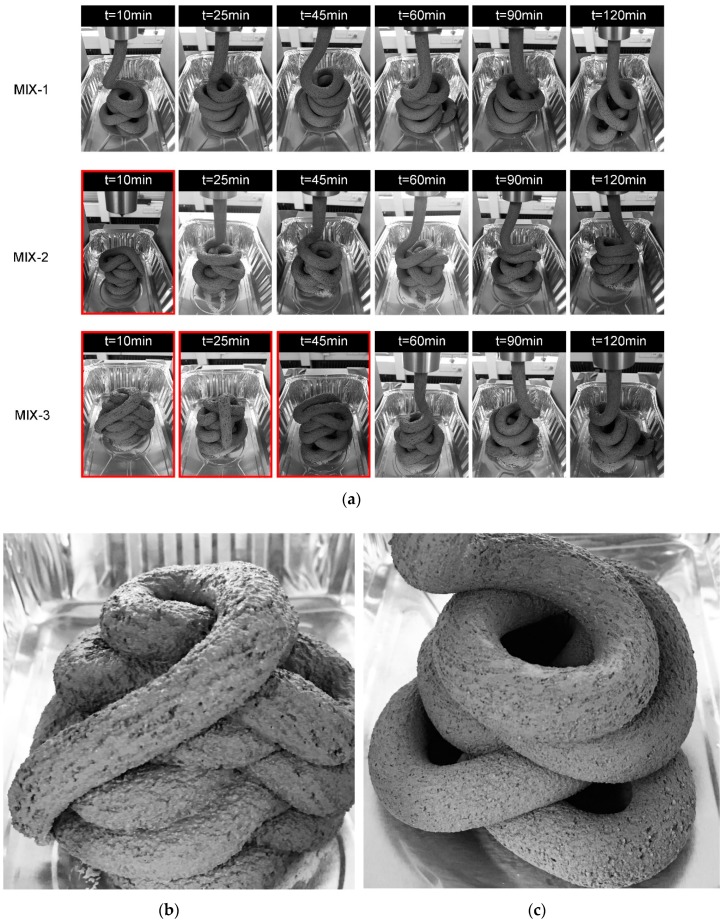
(**a**) Extruded filaments of mixtures MIX-1, MIX-2, and MIX-3 at different ages. The images with the red frame represent that the extruded filaments have a relatively weak shape stability, compared with others. The shape stability was evaluated by visual inspection. (**b**) An example of a relatively weak shape stability (MIX-3 at the age of 10 min). (**c**) An example of a good shape stability (MIX-1 at the age of 10 min).

**Figure 9:**
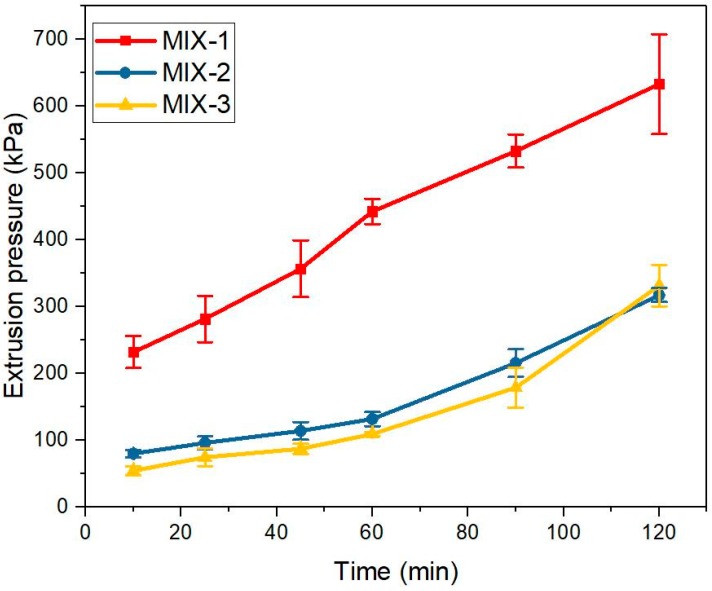
Comparison of the extrusion pressures (under 0.25 m/s of the piston moving speed) between mixtures MIX-1, MIX-2, and MIX-3 at the age of 10, 25, 45, 60, 90, and 120 min. The relative standard deviation (RSD) of the extrusion pressure is under 20%.

The extruded filaments of mixtures MIX-1, MIX-2, and MIX-3 are illustrated in Figure 8. Based on visual inspection, MIX-1 had a better cohesion at all tested ages. For mixtures MIX-2 and MIX-3, a good shape stability could be found after the age of 25 min and 60 min, respectively. The average extrusion pressure (*P*) under 0.25 mm/s of piston rate was calculated using Equation (5). As shown in Figure 9, all curves demonstrated a steadily increasing trend with time. Extrusion pressures increasing with time might be attributed to the loss of workability of fresh cementitious materials. MIX-1 showed the highest extrusion pressure at all tested ages. The extrusion pressure of MIX-1 was more than twice of MIX-2. The test results of mixtures MIX-2 and MIX-3 were very close. Within the initial 90 min, the extrusion pressure of MIX-2 was a little higher than MIX-3. However, MIX-3 showed a slightly larger result at the age of 120 min. Compared with MIX-2, the initial setting time of MIX-3 (129 min) was very close to 120 min. The stiffness development of fresh MIX-3 might be accelerated from 90 min to 120 min. Overall, increasing a certain amount of VMA could increase the extrusion pressure and enhance the shape retention of the extruded filaments in the first 2h.

### 3.2. Extrusion Pressures with Different Material Flow Rates

**Figure 10:**
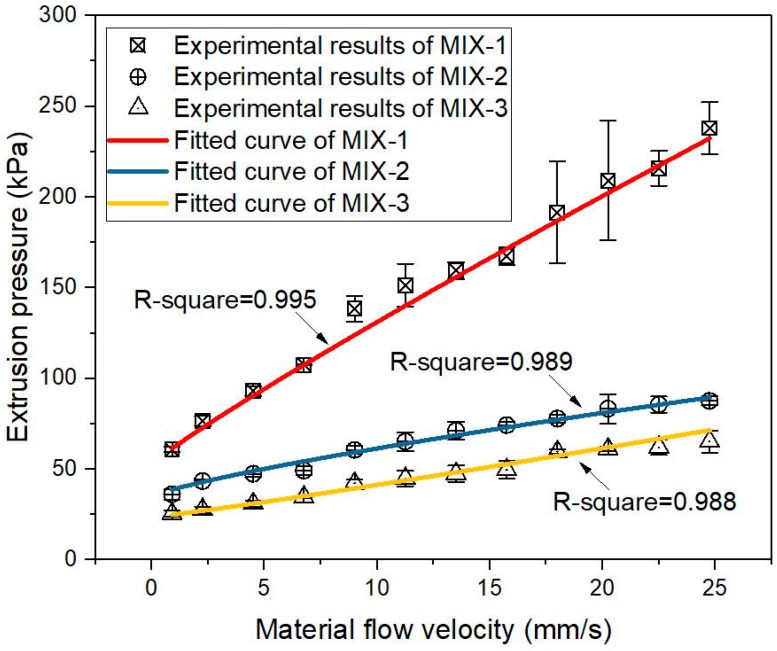
Experimental results of the test – extrusion pressures with different material flow rates and the fitted curves by using the Basterfield et al. model (Equation (2)). The RSD of extrusion pressures was under 20%.

**Figure 11:**
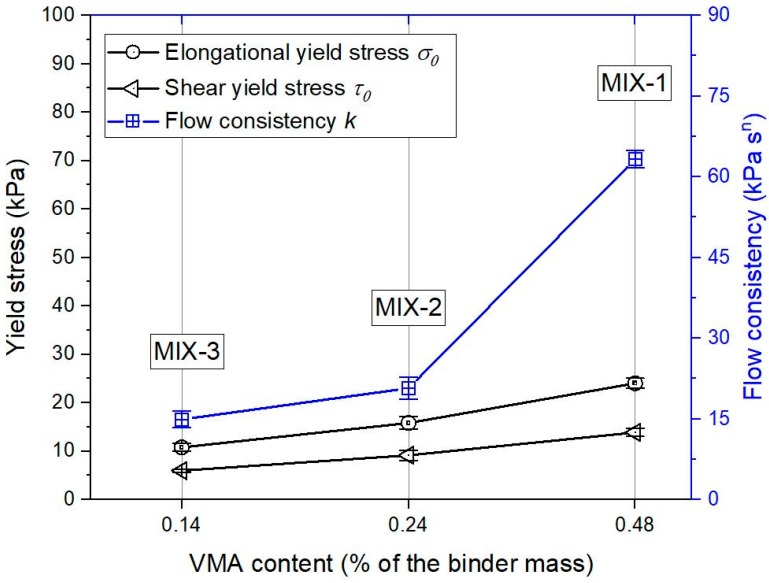
Fitted results of the elongational yield stress *σ_0_* and flow consistency *k*. Shear yield stress *τ*_0_ was obtained by the Von–Mises criterion Equation (3). The RSD of *σ_0_*, *k*, and *τ*_0_ was under 12%.

The similar data process with Section 3.1 was conducted initially. The extrusion force of mixtures MIX-1, MIX-2, and MIX-3 were collected in the measuring zone and transferred to the average extrusion pressures, under various piston speeds. The test results are plotted in Figure 10. The analytical model (Equation (2)) was fitted to the experimental results, by using the non-linear least squares regression analysis in the Originlab, to figure out the material flow parameters including *σ*_0_, *k*, and *n*. Figure 10 demonstrates the fitted curves of mixtures MIX-1, MIX-2, and MIX-3. As the R-squared value in all fitted curves was quite high at about 0.99, the correlation between experimental results and Basterfield et al. model was excellent. According to Equation (3), the shear yield stress *τ*_0_ of mixtures MIX-1, MIX-2, and MIX-3 were determined. The obtained material flow parameters, including *σ*_0_, *k*, and *τ*_0_ are plotted in Figure 11. It could be found that the mixture with a higher VMA content showed a larger elongational yield stress *σ_0_*, flow consistency *k*, and shear yield stress *τ*_0_.

The up-limit of the pump of 3D concrete/mortar printer might be easily reached by pumping and extruding MIX-1. As shown in Figure 10, MIX-1 with 0.48% of VMA (of the binder mass) exhibited a significant high extrusion pressure, under higher extrusion rates. Figure 9 illustrates that the extruding pressure of MIX-1 was continuously increased in the first 2h. For MIX-3, it contained the minimum VMA dosage (0.14%), and showed the smallest extrusion pressure among all mix designs (see Figure 9 and Figure 10). However, according to Figure 8, the stable shape of extruded filaments could not be found until the age of 60 min. Therefore, MIX-2 might be the most suitable mix design for extrusion-based 3DCP in this study. Relatively small extrusion pressures were required at different ages, or under different extrusion rates. It showed a good shape stability, since the age of 25 min.

## 4. Conclusions

This paper attempted to investigate the effect of VMA on the extrudability of limestone and calcined clay-based cementitious materials for 3DCP, by using a ram extruder. Three mix designs with different dosages of VMA were proposed. Mixtures MIX-1, MIX-2, and MIX-3 contained 0.48%, 0.24%, and 0.14% (of the binder mass) of VMA, respectively. All mix designs were initially tested to explore the changes of the extrusion pressure with different ages (t = 10, 25, 45, 60, 90, and 120 min). The shape stability of the extruded filaments was also evaluated. Moreover, the analytical relationship between the extrusion pressures and the different material flow rates was illustrated by using the Basterfield et al. model. Overall, the influence of VMA could be summarized as follows:

- The extrusion pressure of all mix designs exhibited an increasing trend with time. At the same tested age, the extrusion pressure (under 0.25 mm/s of piston speed) was increased by increasing the dosage of VMA. The shape retention of extruded filaments was enhanced as well. MIX-1 had a better cohesion at all tested ages. For mixtures MIX-2 and MIX-3, a good shape stability was found after the age of 25 min and 60 min, respectively.
- Under the same extrusion rates at the age of 10 min, the mixture with higher VMA contents showed higher extrusion pressures. The correlation between experimental results and Basterfield et al. model was excellent (R-squared: 0.99). Increasing the dosage of VMA from 0.14% to 0.48% could increase the elongational yield stress *σ_0_*, the flow consistency *k*, and the shear yield stress *τ*_0_.
- Among all mix designs in this study, MIX-2 might be the most suitable mix design for extrusion-based 3DCP. It not only had relatively small extrusion pressures at different ages or under different extrusion rates, but showed a good shape stability since the age of 25 min.

However, to determine the optimal VMA dosage for limestone and calcined clay-based cementitious materials for 3DCP, additional work is still required:

- The differences between the ram extrusion and screw extrusion have been illustrated by Perrot et al. [27]. Most of the pumps for 3DCP are based on screw extrusions. It is essential to evaluate the extrudability of mixtures MIX-1, MIX-2, and MIX-3, by a screw extrusion pump. The correlation between the ram extruder and the screw extrusion pump needs to be investigated.
- Buildability is also an important constraint for developing 3D printable cementitious materials. Since the HPMC-based VMA could retard the initial setting time and hydration of cement, the early-age strength development might have been affected by using a higher dosage of VMA in the mixtures. Thus, the effects of VMA on early-age strength development of mixtures MIX-1, MIX-2, and MIX-3 are worth to be explored.

## Figures and Tables

**Table 1:** The chemical composition of the dry components in the mix designs.

Oxide (wt. %)	SiO_2_	Al_2_O_3_	CaO	Fe_2_O_3_	K_2_O	Other	Total
PC	17.4	4.1	68.7	2.8	0.6	6.4	100.0
CC	55.1	38.4	0.6	2.6	0.2	3.1	100.0
LF	0.2	0	39.6	0.1	0	60.1	100.0

**Table 2:** Mix designs of the fresh mortar for extrusion by mass ratio to the binder (the binder by mass—Portland cement (PC) + calcined clay (CC) + limestone filler (LF) = 1; the content of PC—331 kg/m^3^).

Type	PC	CC	LF	Sand	Water	SP	VMA
MIX-1	0.4	0.4	0.2	1.5	0.3	0.02	0.0048
MIX-2	0.4	0.4	0.2	1.5	0.3	0.02	0.0024
MIX-3	0.4	0.4	0.2	1.5	0.3	0.02	0.0014

**Table 3:** Piston moving velocity and material flow velocity at the orifice.

*V_0_* (Piston Speed in mm/s)	*V* (Material Flow Velocity at the Orifice in mm/s)
0.10	0.90
0.25	2.25
0.50	4.50
0.75	6.75
1.00	9.00
1.25	11.25
1.50	13.50
1.75	15.75
2.00	18.00
2.25	20.25
2.50	22.50
2.75	24.75
